# Critical Remarks on the "Plea of Insanity," &c.

**Published:** 1855-04-01

**Authors:** Richard Poole

**Affiliations:** Fellow of the Royal College of Physicians, Edinburgh, formerly Superintendent of the Royal Lunatic Asylum, Montrose.


### CRITICAL REMARKS ON THE "PLEA OF INSANITY," &c.

BY RICHARD POOLE, M.D.,

*Fellow of the Royal College of Physicians, Edinburgh, formerly Superintendent of the Royal Lunatic Asylum, Montrose.*

"NEXT after minors," says Baron Hume, commencing this portion of his justly esteemed work, "we may attend to the case of those unfortunate persons who have to plead the more miserable defence of idiocy or madness, which, if it is not pretended, but genuine, and is withal of the due degree, and is fully proved, brings the act to be the same as that of an infant, and has equally the privilege in all cases of an entire exemption from any manner of pain; '*Cum alterum innocentia concilii tuetur, alterum fati infelicitas excusat.*' (L. 12 ad Legem Corneliam de Sicariis.)"

Observe—if *genuine*—of *the due degree*—and *fully proved*—implying serious obstacles in the way of sufficient legal excuse; and these are specially enforced —more particularly on one main point. "I say," continues Baron H., "where it is fully proved, and is of the due degree: For if reason and humanity enforce the plea in these circumstances, it is no less necessary to observe such a caution and temperament in the application of it, as shall hinder it to be understood that there is any privilege of mere weakness of intellect, or of a strange and moody humour, or of a crazy and capricious or irregular temper and habit"—the Commentator expressly declaring, "*None of these things*"—namely, "mere weakness of intellect," "a strange and moody humour," "a crazy and capricious or irregular temper and habit"—"*either are or ought to be law.*" In plain words, weakness of intellect, with the adjuncts enumerated, can form no defence in the eye of law, which, according to Hume, speaking decidedly as an authority, is in this matter consistent with reason and humanity.

We shall see how he endeavours to make out a position equally desirable and important. "Because," are his words, referring to the peculiarities mentioned, "such constitutions are neither exclusive of a competent understanding of the true state of the circumstances in which the deed is done, nor of the subsistence of some steady evil passion, grounded in that situation, and directed to a certain object. To serve the purpose, therefore, of an excuse in law, the disorder must amount to absolute alienation of reason, '*ut continua mentis alienatione, omni intellectu careat*'—such a disease which deprives the patient of the knowledge of things about him, and of the discernment of friend from foe, and gives him up to the impulse of his own distempered fancy, divested of all self-government or control of his passions"—absolute alienation of reason—the want of all intellect—the subversion of every faculty to perceive the plainest distinctions among persons and things—a total surrender to imagination unguided and unchecked by judgment—the unmodified supremacy of animal propensities. These alone, according to Hume, and in his phraseology, are and ought to be law—that is, constitute the due degree of insanity required—and which should be required—by law, in order to "the privilege of an entire exemption from any manner of pain"—or to realize the maxim "*fati infelicitas excusat.*"

Taking for a moment this view of the state of a patient reckoned not amenable to penalties, and seeing that nothing short of it is held adequate to exemption, one might think it rather strange that Hume immediately afterwards expresses a doubt "whether it should be added to the description, that he must have lost all knowledge of good and evil, right and wrong;" and, farther, to find the admission, that "this is a more delicate question, and fit perhaps to be resolved differently, according to the sense in which it is understood." But, in the first place, let us see how two or more differences in the meaning of the question are made out. "If," says Hume, "it be put in this sense, in a case, for instance, of murder—Did the pannel know that murder was a crime? Would he have answered on the question, that it was wrong to kill a neighbour? This is hardly to be reputed a just criterion of such a soundness as ought to make a man accountable in law for his actions. Because it may happen a person to answer in this way, who yet is so absolutely mad as to have lost all true observation of facts, all understanding of the good or evil intentions of those who are about him, or even the knowledge of their persons." This is the first sense, according to Hume, then, in which the question may be put respecting the *admissible* insanity of the individual; and it follows, from the *quasi* answers supposed, that, though labouring under "absolute alienation of reason," a deprivation "of the knowledge of the true position of things about him," and of "the discernment of friend from foe," he may nevertheless be able correctly to answer—of course, to understand—certain questions both in morality and in law; or, in other words, it follows, that "absolute alienation of reason," &c., is not essential to the requirement of law, namely, that insanity, in order to be effectual as a bar against penalties, must amount to such a degree as implies that totally defective condition of mind. Now, pray do the inconsistencies and contradictions of medical men, as to which judges and lawyers are so apt to be facetiously critical, ever reach the magnitude of this self-destructive statement, one, be it remarked, not by any means confined to the Commentator under review? But let us advance to a second sense of the question propounded by him.

"If it be put in this other and more special sense, as relative to the very act done by the pannel, and the particular situation in which at that time he conceived himself to stand (observe—*conceived himself to stand*): Did he, as at the moment of doing that thing, understand the evil of it? Was he impressed with the consciousness of guilt, and fear of punishment? it is then a pertinent and material question, but which cannot to any substantial purpose be answered, without taking into consideration the whole circumstances of the situation." Doubtless—and so, therefore, it appears a loophole is provided should there be occasion for it—entire alienation is consistent or not, as the case may be, with the presence of such and such faculties; consequently the requirements of law may or may not be insisted on in different circumstances! This dubiety—in truth double-dealing, gloss it as we choose—is made apparent, notwithstanding the pains to conceal it. "Every judgment in the matter of right and wrong," continues Hume, "supposes a case, or state of facts, to which it applies." Yes, certainly, but what then? He answers thus, quite at variance with the fundamental position: "Though the pannel have that vestige of reason (previously supposed to have been completely exhausted or lost) which may enable him to answer in the general (an impossibility under the premises) that murder is a crime, yet if he cannot distinguish his friend from his enemy, but conceive everything about him to be the reverse of what it really is, and mistake the illusions of his fancy for realities, with respect to his own condition and that of others, '*absurda et tristia sibi dicens atque fingens*,' these remains of intellect (observe again, *remains*, notwithstanding 'absolute alienation') are thus of no use to him towards the government of his actions, nor in anywise enable him to form a judgment upon any particular situation or conjuncture of what is right or wrong with regard to it." (More especially, it would appear in the language of Hume.) "If he does not know the person of his friend or neighbour, or though he do know him, if he is possessed with the vain conceit that he is come there to destroy him, or that he has already done him the most cruel injuries, and that all about him are engaged in one foul conspiracy to abuse him, as well might he be utterly ignorant of the quality of murder. Proceeding, as it does, on a false case, or conjuration of his own fancy, his judgment of right and wrong, as to any responsibility that should attend it, is truly the same as none at all. It is, therefore, only in this complex and appropriated sense, as relative to the particular thing done, and the situation of the pannel's feelings and consciousness on that occasion, that this inquiry concerning his intelligence of moral good or evil is material, and not in any other or larger sense."

Now, while a medical man will readily assent to all this, as indicating by much the most frequent case of insanity, he must be allowed to demur in respect to its harmony with the general definition of the malady laid down in law, and with the amount or kind of it demanded by law as an adequate plea for "the privilege of an entire exemption from any manner of pain." Look at the suppositions—the admitted premises—here detailed. The pannel has a vestige of reason, which enables him to answer in the general (of course understanding) that murder is a crime: he has remains of intellect (which, consequently, is not absolutely alienated), and likewise a judgment of right and wrong (though said to be "truly the same as none at all"): but, at the same time, it is predicated of him that the remains of intellect are of "no use to him towards the government of his actions"—that "he does not know the person of his friend or neighbour," or, though he do know him, he is possessed with "a vain conceit" regarding him—that, from a similar source, he supposes others are engaged in a conspiracy against him; and, in short, that, proceeding on a false case, or conjuration of his own fancy, his judgment is quite nugatory or unavailing, possibly even worse than useless. What is the legal result? Hume does not expressly tell us, but leaves to be inferred pretty clearly. "In this complex and appropriate sense" (beyond a doubt *complex*—but what is to be inferred from the term "appropriate"?) the inquiry concerning the pannel's "intelligence of moral good or evil is material"—that is to say, may lead to his "exemption from any manner of pain." Verily, so it should, and to this extent there is an agreement amongst most medical men. But, then, is it not apparent, contrary to the intimation or authority of the law, that something distinct from alienation of reason—want of judgment or knowledge as to right and wrong—namely, incapacity or unfitness to discern a friend, the conjuration of fancy, a vain conceit of one kind or other, is that alone, or at least mainly, on which exemption from responsibility depends? Is not this really admitted by Hume himself, though it may be covertly, when he refers to "the situation of the pannel's feelings and consciousness" on the occasion—not his degree of reason—as material to the inquiry? In short, is it not clear, according to the premises, that available insanity is constituted less (*negatively*) by the alienation of reason, a deprivation of the knowledge of the true position of things, than (*positively*) by the presence, excess, and supremacy of some thing different from more intellect, such as Hume himself specifies, namely, the "impulse of distempered fancy," "the illusions of fancy," a "conjuration of fancy," "vain conceit," nay, a state of the passions that does not admit control? Of the soundness of this view, experienced physicians can have no doubt, while some writers among them have dwelt on it, as might be shown; here, however, I am confining myself to a statement of what the law on the subject is, as laid down by one competent to expound it. That statement and his preliminary remarks on the practical application of it are, I repeat, singularly at variance, and inevitably issue in a contradiction of sense, if not in plain words. I must insist on this point, and place it in the clearest possible light. We have, then, on the one hand, the broad and perfectly intelligible assertion that, "to serve the purpose of an excuse in law, the disorder *must amount* to absolute alienation of reason" (enforced by the pithy maxim, "*ut continua mentis*," &c.), such a disease as deprives the patient of the knowledge, &c., and of the discernment, &c., and gives him up to the impulse, &c. On the other hand, an admission to the effect, not only that a man capable of correct answers to certain questions, may yet be so absolutely mad as to have lost all true observation of facts, but likewise that, in another case, privileged to the same extent, be it noticed, the pannel, though having a "vestige of reason," "remains of intellect," may derive no advantage from them, find them "of no use to him," even when most required; because, in opposition to their dictates, he is possessed with a "vain conceit," proceeds on a "conjuration of his own fancy," is influenced solely or mainly by "feelings and consciousness," and, altogether, might as well be utterly ignorant of the quality of his actions, and void of judgment to discern right from wrong.

"But to return to the point whence I set out," says Hume, meaning, it is obvious from what follows, the announcement of law. "Our practice has always been governed by the general precept, already mentioned, which admits of no defence short of absolute alienation of reason." Observe—no excuse short of absolute alienation of reason is admissible in the practice of Scotland. Here, then, and before entering on any of the cases adduced in illustration of the practice, while to obviate all cavilling, I may as well determine the precise meaning of the term or terms thus used. Dr. Johnson shall be my guide in the first place. He gives four senses of the word *alienation*, but only two of them are pertinent—namely, 3. "Change of affection," quoting Bacon thus—"It is left but in dark memory, what was the ground of his defection, and the *alienation* of his heart from the king;" and 4. "Applied to the mind, it means disorder of the faculties," as exemplified in a passage from Hooker: "Some things are done by man, though not against, yet without their wills, as in *alienation* of mind, or any like inevitable utter absence of wit and judgment"—really a very fair description of insanity, as insisted on by the law of Scotland—"any like inevitable utter absence of wit and judgment"—*utter* being quite equivalent to *absolute*, that is, according to Johnson's definitions or synonyms, namely, "complete," "unconditional," "not relative," "not limited," "positive, certain, without any hesitation"—one and all, taken in conjunction with the substantive, implying, as above, "utter absence of wit and judgment," total subversion or transference of intellect, entire deprivation of understanding. This, accordingly, is the amount of disease required for a defence in law, as will be presently seen on recurrence to Baron Hume.

"To that purpose," says he, "the interlocutor upon the case of Robert Thomson, indicted for murder, finds it relevant, and allows him to prove, 'That when the fact libelled was committed, he was so furious, mad, and distracted, *as to be totally deprived of his reason and understanding;* reserving consideration as to the effect of what should be found proven, until the verdict should be returned.' " Again, Hume tells us—"The same principle had governed in the conviction of Thomas Gray (July 26, 27, 1773). This man was indicted for murder, by stabbing. It was alleged for him, that he was of very weak intellect, extremely passionate and flighty, addicted to the immoderate drinking of strong liquors, and, on the whole, what between the use of these and natural infirmity, rather a sort of fool or crazy person, and so considered by his neighbours, than a sound man. This account was confirmed by the witnesses upon the trial, several of whom swore to his being drunk when he stabbed, and that he was at all times a weak and passionate creature, and especially (as they expressed it) 'on the *woodish* order when he got drunk.' All this was plainly short of madness in the sense of law, and the jury therefore found him guilty of the murder."

To the same effect, Hume narrates—"The rule is further confirmed by the case of Robert Bonthorn. The charge against him was that of being a smuggler, and having had contraband goods seized in his possession, he, out of revenge, laid hold of an opportunity violently to push the revenue officer over a precipice upon the sea-shore, whereby the man had his thigh-bone broken, and was otherwise injured. The jury 'find the libel proven, and also find that the intellects of the pannel are most remarkably weak, irregular, and confused, and therefore recommend him to the mercy of the court.' He had judgment, nevertheless, to be twice whipped at different places of the county where he dwelt, and for a sum of damages and expenses."

"For the same reasons which weighed in these cases," continues Hume, "the defence of madness is less suspected, and will more easily be received, against a charge of murder, mutilation, or other violent crime, than of those offences, like theft or forgery, which can hardly be executed without art and steadiness of purpose"—these, Baron Hume might have said, being considered evidences that there was no absolute alienation of reason. Accordingly, he exemplifies thus: "I find that in the trial of Thomas Henderson, for horse-stealing (Feb. 22 and March 9, 1731), it was pleaded for him that he was subject to occasional fits of madness. But the libel charged, that he had conducted himself prudently in the adventure, having stolen the horse in the night, and ridden straightway by an unfrequented road to a distance, where he left the horse, under a plausible pretence, and, last of all, sold it, and took a bill for the price. The interlocutor, therefore, did not take notice of this allegation, but repelled in general all the pannel's defences."

In respect to all these instances—the issues of which need surprise no one—I have simply to remark that, while absolute alienation of reason was not even pretended to exist in them, so, on the other hand, they appear, from the evidence adduced, to have been free from those delusions, deceptions, or hallucinations which occur in and characterize the insanity recognised by medical men. I would say of them, consequently, and in the general, that, while they point out the ordinary, and, be it admitted, the correct application of the law, nothing was either presented or alleged in them to indicate its unsuitableness as a common and unmodified rule. Let us then advance.

"But although the distemper must thus be absolute in degree, it is not material that it be also total in respect of time. The quality of the action has no dependence but upon the pannel's state of mind at the time of doing it; so that whether his malady be constant and unremitting, or only return at intervals, still the defence will be equally available, if he was then utterly furious and void of reason." Observe the expression, "*and* void of reason"—not alone "utterly furious." Accordingly, Hume says—"Here I may cite the case of Sir Archibald Kinloch (June 29, 1795), which was that of a person who, having had his senses injured by the acute delirium of a West India fever, was afterwards liable to occasional fits of derangements, though at considerable intervals, and at length, in a state of utter furiosity, had the misfortune to kill his brother. The violence of this fit had only been for a few days before the fact, and he soon after settled into his ordinary condition. Nevertheless, the jury were unanimous to acquit him." I have no doubt they were right; but the case, I imagine, scarcely bears on the matter at issue. Hume follows it by another "in some measure of the same description"—that, namely, of Robert Spence, tried for murder (June 19 and 30, 1747). "The pannel and the deceased, who was a woman and teacher of a school, were inhabitants of different floors in the same building. And it appears that, having risen in his shirt, in the dusk of the evening, and knocked at her door, he, upon opening, instantly rushed in, and uttering some strange and incoherent exclamations against the woman, knocked her on the head with a hatchet or chopping knife, which he brought with him. After despatching her, he ran off to his own house, and when a *posse* assembled to seize him, he suddenly sprang out upon them, and attempted to escape. It was also a remarkable circumstance that, on returning to his house, he had taken offence at a wig-block which stood there, and violently clove it down with the same hatchet; so that it was found all besmeared with the woman's brains and gore. With regard to his symptoms of disorder for some days preceding, they were chiefly these:—a great degree of restlessness, a disposition to ramble through the country at all hours and without an object, incoherent discourse and disorderly behaviour, though without any act of outrage or violence offered to his neighbour. But it was farther proved that, some years before, when employed as a sailor, he had occasionally shown symptoms of derangement, which were aggravated by drinking, so that he had sometimes been confined on board of ship for eight or ten successive days as insane. The jury found it 'proven that the pannel was furious at the time he committed the said murder, but to what degree of furiosity they could not determine.' On which verdict the court ordered him into confinement, until bail should be found by his relations to keep him in a state of safety." I admit all this to have been correct in point of principle.

Hume says—"There is one more case of the same character upon record—that of Jean Blair (March 14, 1781), who, with a hatchet, cruelly mangled and killed her mistress, with whom she had lived some years as a confidential servant; and then, after setting fire to the house, and defacing the effects within it, ran out stark naked, and with her hands bloody, into the street, and gave the alarm of fire to the guard. It was proved that several of her family had been insane, and that she herself had shown symptoms of insanity about ten years before. She was acquitted of the murder, but ordered into confinement." Very justly, I dare say; but the plea of absolute alienation seems to have been lost sight of; while it is important to observe, that the evidence both of family and personal derangement—to what degree does not appear—was allowed to be received.

Baron Hume expressed dubity whether he ought to refer to the same or to another class, the case of Robert Thomson, previously mentioned. "It was in all its circumstances an extraordinary case. The pannel was accused of the murder of George Forrester, committed at mid-day, by knocking him down from his horse with a stone, and cutting his throat with a pen-knife, as he lay on the ground (a muir on the highway from Haddington to Aberlady)."

Pannel was a blacksmith, and had been employed in his trade as usual, that very morning, till ten o'clock. And farther, not more that half an hour before the murder, two persons, who met him on the highway, had spoken to him in passing, without observing anything unusual in his appearance. A few hours after committing the murder he was taken into custody, and, in the afternoon of the same day, when being conveyed to the jail, he had so far recovered as to be sensible of the deed perpetrated; farther, too, he pointed out the precise spot where he had killed the deceased: showed "the innocent blood" (as he called it) on the ground; said that his own would be shed for it; and expressed concern on account of the distress which he would bring upon his father. He also related to the persons who had charge of him upon the way, that the deceased had many times cried for mercy while he was striking him on the ground; but, "I trow (said he) I had no mercy on him, for I believed it was the devil I killed." In the same strain he added that, before meeting the deceased, he had chased the devil through the muir in another shape—"like a man with a whin-cow in his hat," and who suddenly vanished before him in the pursuit. In confirmation of these strange circumstances, it was proved, that the pannel was subject to convulsive fits, especially at full moon; and that he was melancholy after them, and liable to be troubled with religious horrors, so that he sometimes started from his bed in the night, and spoke and acted as if he were grappling with the devil. He had been seized with one of these fits before the murder, but it had not been followed by any extraordinary depression of spirits. The jury returned a verdict finding "no proof of furiosity till after the murder was committed;" as, "indeed, no one was present to judge of his appearance at that time, and recently before he had been in his ordinary condition. Yet, upon the whole circumstances, there was little room to believe him guilty; and in this light the case had been viewed elsewhere, for he received a transportation-pardon." Here Hume obviously points to Crown interference, because sentence of death must have followed such a verdict—neither furiosity at the time nor absolute alienation of reason having been proven; and yet, I presume to maintain there cannot be any hesitation, among physicians at least, to assert the man's insanity—though, very clearly, it was not without "method," or, in reality, what he thought to be a good object. The absurdity of insisting on a strict interpretation of the law, requiring the sole negative quality—if I may so call it—as an excuse, is palpable in this very instance, were there no other on record. How long will it be ere a conviction shall predominate among lawyers and juries, that, in many cases—perhaps the majority—horrid deeds of the kind have proceeded from the invasion of disease, in which, nevertheless, what is called reason was not at all impaired, not to say annihilated? That the case of Thomson was extraordinary, as Hume states, will be admitted by those only who are not much conversant with the phenomena of insanity, and, particularly, by such as confine their views to the dogmas of merely legal men. But, passing by for the present what experienced physicians must think of it, we come to what Hume represents as "still more strange than any malady of this kind." "There have," he tells us, "even been instances of sudden and, in a great measure, unaccountable frenzy, and which, though excessive for the time, quickly subsided, and never again returned." There *have been*, beyond a doubt, and there will be, in spite of legal exactness. My wonder, and that of others in concurrence, is that, notwithstanding such exactness, Hume should have found occasion to add—"in these, too, the plea of insanity has been sustained." He goes on in proof—"A history of this sort is related by Sir M. Hale; that of a woman who was tried at Aylesbury, in 1668, for the murder of her own child. She was a married woman, and of undoubted good fame and virtuous deportment. But not having slept for some nights after her delivery, and, by this and other disorder of her person, having fallen into a sort of delirium, and being left alone, she killed her infant; which, presently after, she showed to some persons who came in, and told them she had done it. She was instantly carried to jail, where, in a little, she fell into a deep sleep, and on awaking was found to have recovered her senses, and marvelled much how, or on what account, she had come there. The jury very justly found her not guilty of the murder."

It is gratifying to see such an opinion—"very justly;" but what might have been the result had the poor woman been unmarried, of bad fame, and in any degree vicious? Sudden frenzy, we may rest assured, is by no means confined or peculiar to amiable and worthy mothers. I advance.

"Very like this," says Hume, "though the verdict was different, was our case of Agnes Crockat, of the 23rd July, 1756. This woman also had killed her own child. She was an unmarried woman, but had called help to her delivery, and had openly kept and suckled the child for the space of six or seven days. It was sworn to, that, at times, upon the day of the fact, she had been strange in her speech and behaviour, but to which the witnesses had not paid much regard; and being left alone with her child, she laid hands upon and strangled it. She kept it, however, lying openly by her in the bed, till the people of the house returned, and then she showed it them, and told them what she had done, and said that the devil had tempted her!" The case was thus far weaker than the former, in that there was no clear proof of bodily complaint, or of a marked transition from a state of disorder to soundness. The jury, therefore, found her guilty. But "the Royal mercy interposed to prevent her execution." I confess the difference between this and the preceding case—considered medically—does not seem to me so great as to have warranted any in the verdict. Hume, it is true, attaches importance to the existence of bodily complaint; but who knows, I would ask, the amount of it therein, and whether or not there was an equal degree of this in the latter instance? I would further ask, is it quite clear that, according to strict law, any condition of bodily health, not demonstrably affecting reason, to the amount prescribed—that is, absolutely alienating it—ought to influence a jury? We shall be a little enlightened on this point by "a third case of the like character, tried at Jedburgh, in April, 1785," and which issued in a verdict of acquittal.

"The pannel was a man named Robert Coalston, a husbandman or farm-servant. Some years before the fact, he had been struck with lightning, and from that time had occasionally been subject to melancholy and depression of spirits, but not in any remarkable degree, nor such as hindered him to do his business as a servant, and without any sort of tendency to violence or mischief. But of a sudden, and without any new or visible cause, the man became restless and impatient, and having left his master's house in the middle of the night, set a-roaming through the country, without plan or object, and behaving absurdly as he went along, but still without offering violence to any whom he met. In the evening of the next day, he returned to his master's house; and by this time having waxed outrageous, he abused his fellow-servants; assaulted and struck his mistress; and, having suddenly snatched an infant out of her arms, which she had upon the breast, he ran off with it out of the house. A few minutes after, the child and he were found together in an outhouse, the child dead, and dashed to pieces, and the man sitting quietly by it, as quite unconscious of what had passed. He made no attempt to fly or resist, and was carried to jail, where he soon settled into a state of languor and stupefaction; out of which when he recovered, in the course of a few days, he seemed to have no remembrance of these incidents, and suffered great agitation on being told what he had done. (Baron Hume, in a foot-note, says, 'These circumstances do not appear on the record, but are known to me as counsel for the pannel.') The jury found the slaughter proved, but the insanity also proved, and he was ordered into confinement."

It is not to be doubted, that, in this case, Hume attached high consequence to the bodily health as affecting the mental state; and that this materially influenced the jury, who, however, as it strikes me, erred in the complex nature of their verdict. If the insanity were proved, then, technically, and in the eye of law, the slaughter could not be so. A verdict of not guilty—on the ground of derangement—would have been correct. To the statement of this case, Hume adds a short but expressive sentence. "In short, how unaccountable soever to us these visitations of sudden and temporary madness, yet still they are within the compass of this miserable privilege, if the utter alienation of reason for the time be proved." I would desire no other than this passage as an acknowledgment of, at once, inability to explain a physical phenomenon, its validity in excuse of an otherwise unpardonable crime, and the fiction or assumption of law to interpret what is declared to be inexplicable.

Our commentator states only one more instance "of the plea of furiosity sustained," in which "there is anything worthy to be remarked." It is that of James Sommerville, tried in 1704. "This man was one of the town officers of Edinburgh, and had shot a soldier of the town-guard, one of a party which had been sent to seize him, on his becoming outrageous." The chief circumstances alleged in evidence of his insanity are thus narrated: "1. Three or four months before the deed, the magistrates of the city, having observed indications of derangement, had ordered him to keep at home, and appointed him a weekly allowance during his confinement. 2. He had conceived a jealousy of evil intended him, and had applied to the provost for a safe conduct or protection, which was given him, out of indulgence to his humour. 3. About four months before the slaughter, he had called for a sword to kill his brother, who came to visit him. 4. He became slovenly in his person and apparel, instead of careful as formerly, and walked out into the streets with his stockings loose about his heels. 5. He uttered strange and hideous cries in church, and in time of divine service. 6. On the morning of the day libelled, he ran into the street in his shirt, with a drawn sword, and threatened his neighbours. 7. On being committed after the fact, and desired to give up his officer's coat or uniform, he was scurrilous to the magistrate, and desired him 'to go hang himself and his coat.' 8. After commitment, he was so disorderly that it was necessary to confine him in the iron cage. The Court sustained the defence as to his being mad, relevant to assoilzie him from the ordinary pain, the pannel proving that the same morning the defunct was slain he went through the wynd in his shirt, with a drawn sword in his hand, threatening his neighbours, and any other *two* of the qualifications of fury condescended on, except the *fourth*, on which the Lords lay no stress." The jury returned a special and awkward verdict, but which had the effect of saving the pannel's life. It is, indeed, somewhat of a curiosity throughout. "Find it proven—1. That there was a warrant or order given by Baillie Warrander to go to the pannel, and endeavour, by all fair means, to bring him with the said Baillie. 2. We find it proven, that the pannel threatened the persons that desired him to open the door, and go to the said Baillie. 3. We find it proven, that the time they were breaking up the door, Henderson, the defunct, received a shot through the lock-hold of the door, which gave him several wounds in his body, and that he died about twenty-five days thereafter. 4. We find it proven, that when the pannel opened the door, after the shot, he asked, 'How all was?' and he was told by Ferguson, Smith, and Innes, that he had killed a man, to which he made answer, 'God have mercy upon my soul.' 5. We find it proven, that some months before this fact, like the pannel had acted a furious or mad man. 6. We find it proven, that after the fact was committed, the pannel gave Baillie Warrander scurrilous language. And, lastly, finds proven, that at the time the shot was given, and the door opened, that Ferguson, the officer, and soldiers, found the pannel and his wife together."

It seems quite evident, from the fifth point in this strange verdict, that there had been great inattention to the real and clamant derangement of the prisoner, who ought certainly to have been confined at an early period of his malady—but not exactly "*in the iron cage.*"

With this instance Baron Hume closes his series of cases. He then proceeds to discuss what are styled the "Presumptions in cases of Insanity."

In regard to the proof of furiosity, we find it laid down as not disputed, that, "in the case of one who has always been reputed sane, it lies with the pannel fully to establish this, equally as any other defence." "But," he adds, "as to the case of lunacy, or periodical madness, a controversy is agitated in the law books, whether it shall be that the deed was done in furiosity or in a lucid interval." "One thing," he declares, however, "is obvious on this head, that there is no room for presumption, unless in the case, which cannot be a frequent one, that the jury cannot come to a conclusion either way, upon the proof of the pannel's situation of mind, as at the time when the deed was done. For if there be a proof applicable to that period, and if it either establish no symptoms of the disorder, or but very slight ones, it will not defend the pannel that he had formerly (as was the case of Lord Ferrers) and for a length of time been insane." As to "situations of a doubtful character," he says, he can imagine but one "in which it may be reasonable to presume for the influence of disease upon the act." It is that of a person "who ordinarily and for a course of years has been insane, with but few, and short, and imperfect intervals of reason; and more especially," he admits, "this will be just if he is found with the plain symptoms of furiosity upon him recently after doing the deed." He supposes a strong case—namely, that of "one who for years has been confined in a mad-house, if, taking advantage of the occasional liberty which is indulged him, on the faith of any seeming intermission of his fury, he shall make escape from his friends, to whose society he has been restored, and shall kill a person when no one is by to bear testimony to what passes, and shall, afterwards, in the course of the same day, be taken in a state of absolute distraction; he may seem to be within the privilege of this humane construction. In the case, on the contrary, of one whose lucid intervals have been longer and more frequent, the presumption upon a doubtful and defective proof shall be against him, though, by reason of the faulty habit, and the natural suspicion of the lurking vice, where it has once shown itself, weaker evidence may here be admitted to cast the balance than in the trial of one who has never been subject to this affliction. The situation is still more unfavourable to the pannel, if his ordinary condition be that of a sound man, or if his lucid intervals have generally been at stated periods, and of nearly the same endurance, and the deed is done within the regular period of such an interval."

Considering the requirements of law, these are liberal and judicious propositions; while, from the sentiment following them, one might infer that Baron Hume, aware of their value, did not feel assured of their adequacy to afford due security in circumstances of doubt. "But truly," are his words, "it is a vain attempt to compress within a few short maxims all the possible varieties and combinations of these miserable disorders. And, on the whole, it will be much safer to conclude, that if ever so ambiguous a case shall happen, the question will rather be fit for the consideration of the jury, on the whole history and circumstances of the particular malady, as detailed in evidence to them, than for the resolution of the court, as matter of law, by any general rule." Admitting, gratefully, the justice and the mercifulness of this reflection, I submit that the question, or inquiry, touching a matter of fact, is exceedingly important to the community at large—namely, have the proceedings of our courts, and the modes of charging juries, together with the summing-up of evidence, usually been such as actually to bring decisions within the range of common sense and reasonable sympathy? My experience and observation do not, it is very certain entitle me to make reply to such a comprehensive query; but I am grieved to declare, most unequivocally, that, on more than one occasion, it has been my unhappiness to witness, within this sacred province, a tone and spirit which I should have regretted to find in the lowest police-office.

There only remains, in this department of Hume's work, what relates to the judgment in cases of insanity, and this, being almost purely technical, will not detain me long.

The result of a verdict, "finding the defence of furiosity proved," cannot well be any other than the entire acquittal of the pannel—"*cum satis furore ipso puniatur.*" According to Hale, this must equally hold true, "of whichever kind the madness be; whether it be attended with rage, fury, and tempestuous violence, or is only such as takes away the use of reason and memory, and leaves the person in a state of imbecility and stupor; in which, if, as a machine, he do any evil, though without *impetus* or rage, it is not a proper act of his, for which he can be accountable in law." One can scarcely help feeling not a little surprised at such a large concession, adopted after the strictness of interpretation previously contended for; and equal difficulty is there in avoiding the opinion, that it has been forced out from a discovery of the inconsistencies between strict law and some decisions said to be founded thereon. Surely, if the result of such a verdict as is supposed hold *equally true*, "of whichever kind the madness be," the definition of that malady must be taken more largely than is usual; and, moreover, a greater variety and latitude of evidence in support of a plea to that effect, than seems to have always been allowed, must be tolerated in court.

"As to the inferior degrees of derangement or natural weakness of intellect," continues Baron Hume, "which do not amount to madness, and for which there can be no rule in law, the relief of these must be sought either in the discretion of the prosecutor, who may restrict his libel to an arbitrary pain, or in the course of application to the king for mercy. Yet I find, in one case—that of Sommerville—though perhaps not to be approved of as a precedent, a middle course was taken, by absolving the pannel from all corporal pain, but decreeing for a fine to the fiscal, and asythment to the widow and children of the deceased." In both points here mentioned, Hume, I should believe, is perfectly correct—on the supposition that his general view of what constitutes madness, as distinct from "the inferior degrees of derangement," is so.

"One matter, however, there is," concludes Hume, "for which, by just and uniform custom, the Court take order by their sentence, except in those rarer cases of delirium from fever, or other bodily disease, for which an undoubted momentary cause can be assigned;—I mean the providing of security to the public, and to the pannel himself, against the danger of his malady, if unhappily he shall again be afflicted with it. To this end, in the case of Sommerville, the Court appointed him to be confined in the house of correction, 'never to be liberated therefrom, but upon a certificate under the hands of the magistrates and two known physicians, that he has convalesced, and become sound in his judgment.' But more ordinarily the course has been to qualify the order of confinement by a humane provision, allowing the magistrates or keeper of the jail to deliver over the pannel to such relation or other person who shall find sufficient bail in the books of adjournal, to the satisfaction of the Court, and under such penalty as they shall appoint, to keep and detain in safe custody for the future. Deliverance was given to that effect in the case of Robert Spence, in 1747; of Jean Blair, in 1781; of Robert Coalston, in 1785; and of Gordon Kinloch, in 1795; in which last case the penalty of the bail-bond was ten thousand pounds." I do not here advert to later modifications of the provision in question.

Thus, then, I terminate my extracts from that portion of Baron Hume's work which relates to the plea of insanity as having, equally with the state of an infant, the privilege, in all cases, of entire exemption from any manner of pain or penalty. This portion is characterized by the author's usual industry, good sense, unimpassioned sobriety of thought, candour, and homely, unaffected style. I imagine, notwithstanding these properties, that it displays less satisfaction in his own mind than he seems to have entertained when viewing generally the criminal jurisprudence of Scotland, together with less precision and conclusiveness of opinion than he elsewhere manifests, in either supporting or at all dissenting from—he rarely censures—the dicta of judges and the verdicts of jurors. If right in this estimate, I should feel small difficulty in accounting for and sustaining it. Seeing the imperative requirement, and bound, simply as a commentator, to maintain it—namely, that "to serve the purpose of an excuse in law, the disorder must amount to absolute alienation of reason—to a disease which deprives the patient of the knowledge of the true position of things about him," &c.; in fact, such an amount and kind of derangement (fully proved, too) as very rarely occurs, even in the most extensive practice of medical men, and, accordingly, cannot often be borne out by evidence in any court whatever; seeing this, I say, on the one hand, and being aware, on the other, of some, at least—indeed, many—of "the possible varieties and combinations of these miserable disorders," under which, though there may be a "vestige of reason," sufficient to enable a man "to answer, in the general, that murder is a crime," he nevertheless "cannot distinguish his friend from his enemy," but "conceives everything about him to be the reverse of what it really is, and mistakes the illusions of his fancy for realities"—under which, again, though there may be "remains of intellect," they are nevertheless of "no use towards the government of actions," nor "in anywise to enable a man to form a judgment upon any particular situation or conjuncture"—under which (the enumeration is about half completed), though there may be "a judgment of right and wrong," it is "truly the same as none at all"—under which, though there may be "intelligence of moral good or evil," yea, and discernment both of persons and things, nevertheless, a "vain conceit," an unfounded suspicion, the belief of "a false case," the "conjuration of fancy," shall predominate in and overrule "feelings and consciousness;"—knowing, besides, the effects and consequences of certain injuries and ordinary maladies, frequent instances of "sudden and, in a great measure, unaccountable frenzy," "visitations of sudden and temporary madness;" lastly, being alive to the cogent fact that all these and other phenomena may and do exist in innumerable degrees of intensity, under innumerable diversities of circumstances, with innumerable modifications of the mental faculties—themselves involving mysteries, even in the healthy state, as innumerable;—what wonder is it, can any man ask, that the *prejudiced* spectator of their occurrence, though faithful historian of their reception in court, and scrupulous annotator on their fate, should be perplexed by conflicting representations, unable to reconcile them to one antiquated dogma, and much more anxious to furnish other men with a convenient mode of avoiding distinct judgment on them, than happy in divesting himself of a conviction that in many cases this, if actually pronounced, must either outrage humanity, or carry what he styles a "miserable privilege" too far? For my own part, I doubt greatly if Baron Hume ever suspected the true source of the dilemma, obstacle, embarrassment, confusion, and perils, which honest and compassionate juries must frequently encounter—encounter, too, without aid, or guidance, or mitigation, till new light break in upon the law itself—an effulgence from science and philosophy to which, I may safely affirm, some of its administrators have manifested anything but a docile or even a self-becoming spirit.

Should I be permitted to follow up this brief review by similar productions, I shall endeavour, not without hope, to engage the public mind and sympathy in a cause which, though perhaps at first sight ungracious or positively repulsive, will be found, on better acquaintance, as worthy of regard as it is beset with difficulties. That these may be overcome by united and persevering efforts is at once the reason for, and the encouragement of, my own individual labours.

Baron Hume's main proposition, given as preliminary, is in these words—"How clearly soever a crime may be proved to have been committed, there may be circumstances in the situation of the pannel which prevent him from being the fit object of punishment. He may be insane at the time of the trial, or he may have been so at the time of the acts in question." This is an admitted and an indisputable point. I proceed then at once to the nature of the defence founded thereon. "If insanity," says Alison, "be of that complete and perfect kind which entirely overpowers the reason, and takes away from the pannel the power of distinguishing right from wrong, or knowing what he is doing, it forms a complete bar to any criminal prosecution; and the pannel is ordered to be disposed of in such a way as to prevent his being hurtful to others in time to come." Observe—"*entirely* overpowers the reason, and *takes away* the power of distinguishing, &c., or knowing what he is doing"—clear and strong terms, which I believe admit of no doubt whatever. "But," says Alison, "several nice and delicate questions arise as to the degree of insanity which, in law, have this effect." Now, not to dwell on the seemingly ungrammatical expression here used—the relative "which" applying to "questions," if the verb "have" be right; whereas, in reality, as I apprehend, "the degree of insanity which in law *has* this effect" is that about which "nice and delicate questions arise." I say—not to dwell on this trivial error—is it not rather strange that, the major proposition or definition being limited to "that complete and perfect kind of insanity which entirely overpowers reason, and takes away the power of distinguishing," &c., there should be any question at all as to the degree required in law? Most certainly questions may and do arise as to matters of fact; but, in respect to law simply, one would imagine there ought to be none, unless—if even this were of any consequence—completeness and perfection admit of degrees; and it were possible that, when or after a power is taken away from a man, it should still remain in him? We shall see how this matter—apparently incomprehensible, and involving a contradiction—is attempted to be cleared up.

Alison's first special proposition is as follows:—"To amount to a complete bar to punishment, the insanity, either at the time of committing the crime, or of the trial, must have been of such a kind as entirely deprived him of the use of reason, as applied to the act in question, and the knowledge that he was doing wrong in committing it." Here we have the former position, but modified in a peculiar manner, "entirely deprived of the use of reason," as "*applied to the act in question;*" and, accordingly, a distinction may hence spring up. Alison thus comments on the statement: "Though law requires, as a complete defence against a criminal prosecution on the ground of insanity, that the pannel should have laboured, at the time of committing the act, under a complete alienation of reason (referring to Baron Hume), yet it is not to be understood that this means either that he was altogether furious, or did not understand the distinction of right or wrong." Indeed! In the face of a definition which insists on the reason being "entirely overpowered," the power of distinguishing right from wrong being "taken away," or, as stated immediately above, "a complete alienation of reason!" And yet judges and lawyers will tease, sneer at, medical men, for their inconsistencies and conflicting statements! But Alison justly remarks: "Cases of that extreme kind (the absolutely alienated) very seldom occur, and certainly much more unfrequently than the instances in which the pannel's state of mind has been such as to render him not a fit object of punishment." Farther, he adds, with entire truth: "It is very seldom that a mad person is either deprived of the power of knowing what he is doing, or of reasoning and conversing on its different subjects (what is meant by the pronoun "its" may be inferred), or of understanding the distinction between right and wrong, in the *general* case and with reference to other persons." But then it would seem, notwithstanding repeated assertions, the requirement of law *does not* tally with the limitation here adduced, unless, indeed, we can prove an impossibility, namely, that "entire deprivation of the use of reason," its "absolute alienation," is consistent with the state of a man, who, though reckoned mad—excusably so—for this is the state supposed—nevertheless possesses the power of knowing what he is doing, of understanding the distinction between right and wrong—yea, too, and of reasoning and conversing thereon. Alison, it is true, adds, "in the *general* case, and with reference to other persons;" but this qualification, very obviously, merely shifts the position of the error perpetrated in the law itself, and leaves intact the difficulty of applying it to numerous cases. We shall find the same incongruity hereafter, as carried out in practice; nay, it is presented in the very next sentence of Alison's *Commentary*. "The great characteristic of insanity, which originates in the general case, is an excessive turning of the mind to its own affairs, consists in an alienation of reason with reference to itself, and in the illusions under which it labours, and the chimeras it has nourished in regard to its own concerns. Few men are mad about others, or things in general; many about themselves. Although, therefore, the pannel understands perfectly (the admission, I need scarcely remark, is strong enough to imply the very reverse of absolute alienation) the distinction of right and wrong; yet, if he labours, as is generally the case, under an illusion and deception, as to his own particular case, and is, thereby, disabled from applying it correctly to his own conduct, he is in that state of mental alienation which renders him not criminally answerable for his actions." Now, granting the accuracy of this description, which is really not far from a true portraiture, as known to experienced men, might we not fairly expect that the terms of the law should be in accordance with it, or, at the very least, that in place of insisting on a *negative*, they maintained the necessity of a *positive* state or condition of mind as characteristic of excusable insanity, namely, such as Alison himself denotes, rather sparingly, indeed, but still intelligibly, "illusions and chimeras, an excessive turning of the mind to its own affairs," madness about one's-self, all, it seems to be admitted, quite compatible with soundness in regard to other persons and things in general?

I do not stop here to animadvert on the curious circumstance, the apparent anomaly (judged of in relation to what law exacts), represented by Alison, when, resting on what he deems the "great characteristic of insanity," namely, "an excessive turning of the mind to its own affairs," he, at the same time, allows that the pannel "understands perfectly the distinction of right and wrong," and yet labours under an illusion and deception as to his own particular case. It does, indeed, seem mysterious that such an excessive turning should be accompanied by, or productive of, disability or inability in other respects. But the difficulty to account for the fact, well known and often exemplified, is neither removed nor lessened by the hypothesis which joins complete alienation of reason with the possession of sound judgment, limited as the latter may be in application. We shall find, accordingly, that a degree of confusion—in truth, a species of contradiction—pervades nearly all the attempts at reconciling legal decisions to the actual facts of cases on record. Alison gives a general view of these under a specific form.

"For example, a mad person may be perfectly aware that murder is a crime, and will admit that, if pressed on the subject; but still he may conceive that a homicide he had committed was nowise blameable, because the deceased had engaged in a conspiracy with others against his own life; or was his mortal enemy, who had wounded him in his dearest interests; or was the devil incarnate, whom it was the duty of every good Christian to meet with the weapons of carnal warfare. If, therefore, the accused is in such a situation, that, though possessing a sense of the distinction between right and wrong, he cannot apply it correctly to his own case, and labours under an illusion which completely misleads his judgment, as mistaking one person for another, or fastening a dreadful charge, entirely groundless, on a friend, he is entitled to the benefit of the plea of insanity in defence against a criminal charge" (referring to Baron Hume). Very properly, say I; but, let me ask, on what grounds, in the face of a law demanding proof of absolute alienation of reason; whereas, on the showing of the case, that faculty, so far from being even in abeyance, not to say abrogated, is actually exercised and manifested? I shall be answered, of course, by the remark, Oh, he cannot apply it correctly to his own case. It may be so, I rejoin; but why? Because, adds my opponent, he labours under an illusion which completely misleads his judgment, because he is grossly mistaken in his conception of things, specially and obviously deceived. Not a doubt of it. But, granting this, why is not the decision in his favour placed on the right, the true basis, the illusion, the mistake, the deception, instead of a fiction, a misnomer, nay, a nonentity, "absolute alienation of reason," disproved and utterly contradicted by admitted facts? Let jurists meet this question as they please. Common sense will deem it answerable in one way only, and pronounce the law to be an absurdity.

Alison goes on, still maintaining the interpretation now adverted to. "This principle was well expressed by Dr. Monro, sen., in the case of David Hunter, 13th March, 1801, charged with murder. Dr. M. deponed that he was incapable of judging of *the propriety of his actions*, or of reasoning with propriety upon them; and, in particular, he gave the deponent a strong indication of this, by leading the deponent to believe that he had been led to commit the crime of which he stood accused, by the circumstance of the women whom he was accused of shooting, having *smothered his own mother*, in the presence of a number of persons who had made it up among them; and that the pannel did seem to have any remorse at what had happened, saying repeatedly that the women had shed innocent blood." This statement is followed up by a quotation from Lord Hale. "It is the condition of very many, especially melancholy persons, who, for the most part, discover their defect in excessive fears and griefs, and yet are not wholly destitute of the use of reason; but this partial insanity seems not to excuse them in the committal of any capital offence. Doubtless mad persons who kill themselves are under a partial degree of insanity when they commit these offences; and it is very difficult to define the invisible line that divides perfect from partial insanity; but it must rest upon circumstances, to be duly weighed by the judge and jury, lest, on the one hand, there be an inhumanity towards the defects of human nature; or, on the other, too great an indulgence shown to great crimes."

Partial insanity, that condition in which a man is not "wholly destitute of the use of reason," seems, then, according to Hale, evidently approved by Alison, "not to excuse him in the committal of any capital crime;" "mad persons that kill themselves," are, doubtless, it would appear, in that condition, for, as the former says, they are under a partial degree of insanity when they commit these offences, which, therefore, are without excuse, though almost invariably declared otherwise at inquests; and the line of distinction between perfect and partial insanity, which cannot easily be traced—indeed is invisible—"must rest upon circumstances to be duly weighed," &c., that is, judges and juries must be guided by these, candidly and humanely considered, in coming to a decision in each individual case. Now, in the very instance of Hunter, above given, as to which Dr. Monro is said to have "well expressed" the principle contended for, I think it quite clear that there was no evidence whatever of the man being "wholly destitute of the use of reason;" that, on the contrary, he actually displayed an exercise of it (erroneously, no doubt) in the deed with which he was charged; and that the terms employed by Dr. Monro to denote his condition, besides really affirming this last fact ("he gave a strong indication, &c., by *leading the deponent*," &c.), are by no means equivalent to, or synonymous with, a declaration amounting to the degree required in law as a valid excuse. What does Dr. M. exactly mean? Not surely that the man was totally deprived of judgment or reason—incapable of exercising any whatsoever or in general—but "incapable of judging of the propriety of his actions, or of reasoning with propriety upon them;" and why or how so? Not because of a radical deficiency in, or the absence of, such a faculty, but, as Dr. M. evidently implies, because of a circumstance (true or false matters not) which the man positively believed, and through the still existing influence of which he was exempted from "any remorse at what happened," saying, expressively, "the women had shed innocent blood," and leaving the inference to be made, "therefore deserved to die," another proof of reason in exercise, be it noticed, under a delusion. That delusion, then, it appears, constituted the essence of his malady, and not the want or even the impediment of reason.

Sheriff Alison himself, I suspect, must have occasionally arrived at something like a similar conclusion, and probably, therefore, felt a misgiving as to either the interpretation or the application of the fundamental law. I find, accordingly, that, having detailed the case of Robert Spence (for which see my notes on Hume), terminating in the dubious verdict, "the pannel was furious at the time he committed the murder, but to what degree they (the jury) could not determine," in pursuance of which he was ordered to be confined for life. Alison adds, "it was plain that, though not insane on every subject, he laboured under some hallucination with reference to the object of his violence." So, then, in this instance, it would appear, contrary to Hale's position, "partial insanity" did form an excuse; while, to their credit be it said, the jury "duly weighed" circumstances, and came to a decision no less humane than judicious. Were successors to follow their example, legal or legalised murders would be comparatively "few and far between."

"Of the same character," according to Hume, followed by Alison, without particular remark, further than as expressive of approval ("of course, acquitted," &c.), was the case of Jean Blair (for which see former notes). He comes next to "a more difficult case," and which, he says, "well illustrates the delusions under which insane persons labour," namely, of Robert Thomson. Hume, as will be seen elsewhere, terminated his narrative of it thus, "upon the whole circumstances there was little reason to believe him guilty (meaning, because really insane); and in this light the case had been viewed elsewhere," &c.; to which Alison adds, "there seems little doubt that he was insane at the time of committing it" (the murder).

We have now another similar case, that of Ann Sparrow, autumn, 1829 (of course, not in Hume's first edition), and of which the following are details. She had, it appears, poured vitriolic acid in considerable quantities down the throat of her own child (a girl seven years old), and nearly killed it. After the horrid deed, "she ran into the neighbours' houses in a state of evident derangement, saying that she had killed the devil." Before this, however, she had frequently threatened her own life—expressed a resolution to commit suicide. "The case was proved, as well as the insanity, and she was ordered to be confined for life"—a decision, every one will probably grant, quite unexceptionable. "So also," says Alison, "in a case related by Sir M. Hale," of which, as of some other cases, I have said enough in the previous article.

(*To be continued.*)

